# Nocturnal systolic blood pressure is increased in restless legs syndrome

**DOI:** 10.1007/s11325-016-1333-0

**Published:** 2016-03-18

**Authors:** Mariusz Sieminski, Markku Partinen

**Affiliations:** 1 Department of Adults’ Neurology, Medical University of Gdansk, Debinki 7, 80-210 Gdansk, Poland; 2 Vitalmed Helsinki Sleep Clinic, Valimotie 21, 00380 Helsinki, Finland

**Keywords:** Restless legs syndrome, Blood pressure, Hypertension

## Abstract

**Purpose:**

Restless legs syndrome (RLS) is a frequent sensorimotor disorder characterized by an urge to move the legs, with symptoms appearing during the night and disturbing nocturnal sleep. There is a growing body of evidence that RLS correlates with an increased risk of cardiovascular diseases and hypertension. The aim of this study was to test the hypothesis that patients with RLS have higher blood pressure (BP) during the night than people without RLS.

**Methods:**

We have analyzed polysomnographic (PSG) recordings of 30 patients with RLS and 27 subjects without the sleep disorder. During PSG, beat-to-beat BP measurement was performed.

**Results:**

Patients with RLS have higher nocturnal and sleep-time systolic blood pressure compared to controls (124.4 vs. 116.5 mmHg, *p* < 0.05; 123.5 vs. 116.1 mmHg, *p* < 0.05). There was no noticeable dip in the values of nocturnal systolic pressure of patients with RLS.

**Conclusions:**

Our results support the hypothesis that RLS and hypertension are linked. Thus, we believe patients with RLS require close observation with regard to cardiovascular risk factors.

## Introduction

Restless legs syndrome (RLS) is a sensorimotor disorder with a prevalence in the general population of 5–8.8 % [1] and is characterized by the presence of an irresistible urge to move the legs frequently accompanied by unpleasant sensations in the lower limbs. Symptoms appear at night or during rest and are relieved by satisfying the urge (i.e., moving the legs [2]). Untreated RLS may lead to insomnia and fatigue.

As described in an extensive review by Ferini-Strambi et al. [3], mounting evidence strongly supports the existence of a link between RLS and cardiovascular diseases (CVD) including hypertension. Nevertheless, causality, or even the direction of possible causality, is still far from established. Considering the clinical consequences and prevalence of hypertension on one hand, and the high prevalence of RLS on the other, efforts aimed at clarifying this relationship are easily justified. Cross-sectional population studies have suggested a higher prevalence of hypertension in the population of subjects with RLS [4–6], but other authors have not found the same result [7, 8]. Notably, though, Erden et al. have found that RLS is an independent determinant for abnormal blood pressure (BP) patterns in which BP does not dip at night (non-dipping pattern) [9].

Attempts to explain the relationship between RLS and BP on the basis of the microarchitecture of sleep have focused on the role of periodic limb movements in sleep (PLMS). PLMS are stereotypical, involuntary movements of the lower limbs during sleep, lasting from 0.5 to 10 s, in series with intervals of 5 to 90 s between single movements [10, 11]. An increased number of PLMS is found in approximately 80 % of RLS subjects [12]. Furthermore, PLMS are followed by a significant increase in BP, in both patients with RLS and healthy subjects [13–15]. It is hypothesized that these repetitive increases in BP during the night may lead to the development of more persistent hypertension in patients with RLS.

A possible role of RLS as a risk factor for hypertension or cardiovascular morbidity was analyzed in large epidemiologic projects. The impact of PLMS on blood pressure was assessed in single night polysomnographic studies. RLS and PLMS are frequently considered as a clinical continuum although they are not synonymous. Therefore, publications focusing on the influence of RLS on nocturnal blood pressure are missing. Based on these data, the aim of this study was to test the hypothesis that nocturnal blood pressure is higher in patients with RLS than in subjects without sleep disorders.

## Material and methods

We performed a retrospective analysis of polysomnographic (PSG) recordings and clinical data of 30 consecutive patients with RLS, diagnosed at the Vitalmed Sleep Clinic in Helsinki, Finland. All patients were diagnosed according to the International Restless Leg Syndrome Study Group (IRLSSG) diagnostic criteria [2]. The diagnosis was made by a clinician admitting the patient to the sleep clinic. The signs, symptoms, and diagnosis were noted in the patient’s file. We analyzed the first PSG recordings of the patients, taken before initiation of any form of therapy for RLS. The clinical data were taken from the patients’ files. Those patients that were taking drugs for any other conditions maintained their standard treatment regimens, and it was determined that no therapeutic changes were made during 2 weeks before the PSG. We excluded patients with any comorbid sleep-related breathing disorders (SRBD), such as sleep apnea, since SRBDs have significant influences on BP.

Twenty-seven subjects with no clinical sleep disorder were included as the control group. These subjects were consecutive patients that underwent a somnological diagnostic battery due to subjective complaints of daytime fatigue or sleepiness, and the results of the diagnostic procedures (history, clinical examination, PSG) revealed that no control subjects had any significant sleep disorder. The subjects underwent a PSG recording and were included only if they were not prescribed any drugs potentially influencing sleep architecture. The clinical data of the patients were taken from their medical files.

The examined and control groups were selected to detect a 10 % difference in values of blood pressure with power test 0.75 (sample size, RLS = 30; sampling ratio = 1.1 (RLS/controls)).

Patients underwent a single PSG study. All recordings were performed with the SOMNOscreen plus PSG system (Somnomedics, Randersacker, Germany). Sleep recordings included four EEG leads, two bilateral electro-oculogram leads (EOG), bilateral chin electromyographic leads (EMG), two surface EMG leads placed on the left and right anterior tibialis muscles (recording periodic limb movements (PLMs) in sleep and wake). Respiration was recorded with a nasal cannula, thoracic and abdominal strains, and finger oxymetry. Electrocardiograms were recorded with a single precardial lead. The PSG included beat-to-beat blood pressure measurement determined via measurement of pulse transit time (PTT) [16]. The measurement of blood pressure was continuous and non-invasive and did not disturb the sleep of the patients. The PTT-based measurement of BP was calibrated and then validated against sphygmomanometric (cuff) measurement of BP on the brachial artery, at the beginning of recording.

The PSG recordings were scored according to the American Academy of Sleep Medicine guidelines [17]. The following sleep parameters were calculated: total sleep time (TST); sleep efficiency (SE); latency of stages 1, 2, slow-wave sleep (SWS), and REM sleep; duration of stages 1, 2, SWS, and REM; Sleep Stage Change Index (number of transitions between the sleep stages per hour of sleep); the Wake Index (WI; number of awakenings per hour of sleep), duration of Wake after Sleep Onset (WASO), and PLMS index (PLMSI).

For the assessment of the cardiovascular system, the following values were calculated: mean systolic and diastolic blood pressure (SBP and DBP, respectively), and heart rate (HR) during the “day” (“day” was defined as the time from the start of recording until the “lights-off” moment, and from the “lights-on” moment until the end of recording), during the night (defined as the time between “lights-off” and “lights-on” moments), and during wake and sleep periods.

Values of nocturnal and diurnal blood pressure were compared between patients with RLS and controls. Values of daytime and sleep BP were compared for each group to check whether a nocturnal dipping of BP appears. Patients with RLS were further divided into 2 subgroups: subjects with a significant (>15) PLMSI and subjects without significant PLMSI The cut-off value of PLMSI >15 was chosen accordingly to available literature [18]. Values of BP were compared between those two subgroups. For further analysis of the impact of PLMSI on BP, coefficients of correlation between PLMSI and values of BP were calculated.

The study was approved by the Independent Bioethical Committee for Scientific Research at the Medical University of Gdansk.

## Results

Participants included 30 RLS patients and 27 controls. Their demographic and clinical data are presented in Table 1. The groups did not differ in terms of age or sex.

**Table 1:** Demographic and clinical characteristics of study subjects with restless legs syndrome (RLS) and controls

	RLS (*n* = 30)	Controls (*n* = 27)	*p*
Sex (M/F)	12/18	12/15	NS
Age (mean ± SD, years)	49.0 ± 14.9	44.3 ± 16.3	NS
BMI (mean ± SD, kg/m^2^)	26.4 ± 4.8	25.3 ± 4.6	NS
Hypertension (*n* (%))	5 (16.7 %)	2 (7.4 %)	NS
Coronary heart disease (*n* (%))	1 (3.3 %)	0	NS
Stroke (*n* (%))	2 (6.7 %)	0	NS
Diabetes (*n* (%))	3 (10.1 %)	0	NS
Hypercholesterolemia (*n* (%))	5 (16.7 %)	0	NS

Patients with RLS had shorter total sleep time, lower sleep efficiency, longer sleep latency, longer time of wake after sleep onset, and a higher index of periodic limb movements (PLMS-I). Nineteen patients with RLS (63.3 %) had PLMSI higher than 15. The exact data on sleep parameters are presented in Table 2.

**Table 2:** Sleep parameters of patients with restless legs syndrome (RLS) and controls

	RLS	Controls	*p*
TST (mean ± SD; min)	370.7 ± 87.9	418.8 ± 57.9	0.01
Sleep efficiency (%)	74.5 ± 14.4	84.3 ± 8.8	0.002
Sleep latency (mean ± SD; min)	26.0 ± 25.7	16.5 ± 9.6	0.04
Wake Index	5.4 ± 2.3	4.9 ± 2.9	NS
WASO (mean ± SD, min)	89.0 ± 65.9	54.3 ± 43.0	0.01
AHI (mean ± SD)	1.3 ± 1.4	1.5 ± 1.3	NS
PLMS-I (mean ± SD)	26.9 ± 25.5	12.0 ± 13.4	0.004
PLMSA-I (mean ± SD)	4.5 ± 5.5	2.1 ± 3.7	0.03
Arousal Index (mean ± SD)	17.3 ± 9.5	14.2 ± 5.0	NS
S1 (mean ± SD, % TST)	17.6 ± 6.8	16.0 ± 8.5	NS
S2 (mean ± SD, % TST)	46.2 ± 8.1	49.3 ± 8.5	NS
SWS (mean ± SD, % TST)	17.2 ± 7.4	16.4 ± 8.9	NS
REM (mean ± SD, % TST)	17.9 ± 6.8	18.2 ± 6.1	NS
Nocturnal SVB	19.8 ± 7.8	19.8 ± 6.4	NS
HR (mean ± SD)	59.6 ± 8.4	62.0 ± 9.9	NS

*TST* total sleep time, *WASO* time of wake after sleep onset, *AHI* apnea/hypopnea index, *PLMS-I* periodic limb movements in sleep index, *PLMSA-I* periodic limb movements in sleep with arousal index, *SVB* sympathovagal balance, *HR* heart rate, *SD* standard deviation

Mean values of blood pressure measured during daytime and wake did not differ between the groups. There was a statistically significant difference in mean values of systolic blood pressure measured during nighttime and during sleep (Table 3).

**Table 3:** Mean values of blood pressure (BP) in patients with restless legs syndrome (RLS) and controls

	RLS	Controls	*p*
Systolic BP in wake (mean ± SD, mmHg)	123.0 ± 12.7	121.6 ± 22.5	NS
Systolic BP in day (mean ± SD, mmHg)	128.1 ± 14.3	123.7 ± 14.8	NS
Diastolic BP in wake (mean ± SD, mmHg)	77.9 ± 12.2	74.7 ± 9.4	NS
Diastolic BP in day (mean ± SD, mmHg)	79.0 ± 12.4	75.6 ± 9.7	NS
Systolic BP in sleep (mean ± SD, mmHg)	123.5 ± 14.1	116.1 ± 15.8	0.03
Systolic BP in night (mean ± SD, mmHg)	124.4 ± 14.7	116.5 ± 15.6	0.03
Diastolic BP in sleep (mean ± SD, mmHg)	76.6 ± 10.8	72.2 ± 11.7	NS
Diastolic BP in night (mean ± SD, mmHg)	77.0 ± 10.8	72.5 ± 11.7	NS

In the control group, values of systolic BP during the night and sleep were significantly lower than the values of systolic BP during the day. However, no such difference was observed in the RLS group, nor was it observed for the values of diastolic BP in either group (Table 4).

**Table 4:** Differences between values of blood pressure (BP) measured during the day and the night in subjects with restless legs syndrome (RLS) and controls

	Daytime values	Nighttime values	*p* ^1^	Sleep values	*p* ^2^
Systolic BP—control group (mean ± SD, mmHg)	123.7 ± 14.8	116.5 ± 15.6	0.04	116.1 ± 15.8	0.04
Systolic BP—RLS group (mean ± SD, mmHg)	128.1 ± 14.3	124.4 ± 14.7	NS	123.5 ± 14.1	NS
Diastolic BP—control group (mean ± SD, mmHg)	75.6 ± 9.7	72.5 ± 11.7	NS	72.2 ± 11.7	NS
Diastolic BP—RLS group (mean ± SD, mmHg)	79.0 ± 12.4	77.0 ± 10.8	NS	76.6 ± 10.8	NS

p^1^: *p* values for comparison daytime values vs. nighttime values (*t* test); p^2^: *p* values for

**Fig. 1:**
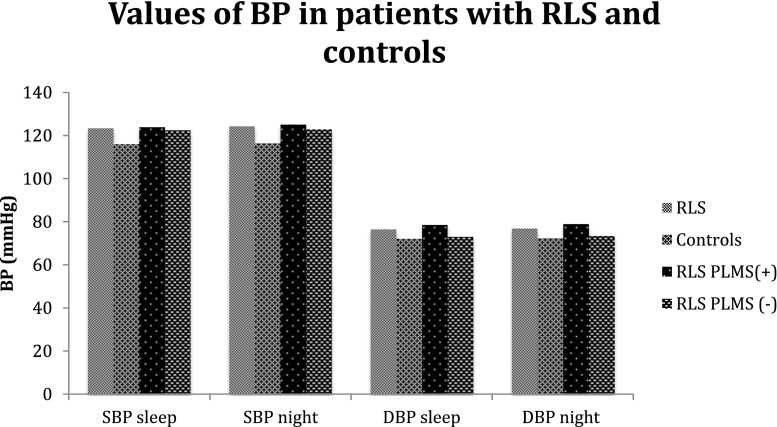
Values of nocturnal BP in patients with RLS and in controls. *BP* blood pressure, *SBP* systolic blood pressure, *DBP* diastolic blood pressure, *RLS PLMS(+)* patients with RLS with PLMSI >15, *RLS PLMS(−)* patients with RLS with PLMSI < 15, *SBP sleep* systolic blood pressure in sleep, *SBP night* systolic blood pressure in night, *DBP sleep* diastolic blood pressure in sleep, *DBP night* diastolic blood pressure in night

There was no significant difference in values of nocturnal BP between patients with RLS with increased PLMSI (>15) and without increased PLMSI (Table 5). The mean values of BP for patients with RLS and increased PLMSI, with RLS and without increased PLMSI, and controls were shown in Fig. 1. There was no significant correlation between PLMSI and nocturnal systolic BP (*r* = 0.24; *p* = 0.07) and nocturnal diastolic BP (*r* = 0.24; *p* = 0.07) in the examined population (Table 6).

**Table 5:** Mean values of nocturnal blood pressure in patients with RLS with PLMSI >15 and PLMS <15

	RLS (+) PLMSI <15 (*n* = 11)	RLS(+) PLMSI >15 (*n* = 19)	*p*
Systolic BP in sleep (mean ± SD, mmHg)	122.6 ± 14.2	124.0 ± 14.4	NS
Systolic BP in night (mean ± SD, mmHg)	123.0 ± 14.1	125.2 ± 15.4	NS
Diastolic BP in sleep (mean ± SD, mmHg)	73.1 ± 10.0	78.6 ± 11.0	NS
Diastolic BP in night (mean ± SD, mmHg)	73.5 ± 10.1	79.0 ± 10.1	NS

**Table 6:** Logistic regression models for relations between total sleep time (TST), periodic limb movements in sleep index (PLMSI) and systolic blood pressure in night (SBP_Night)and systolic blood pressure in sleep (SBP_Sleep)

	OR	95 % CI	*p*
Model 1			
TST × PLMSI	1005	1000–1011	0.0676
SBP_NIght	1.032	0.991–1.074	0.126
Model 2			
TST × PLMSI	1.005	1.000–1.011	0.0625
SBP_Sleep	1.030	0.990–1.072	0.1385
Model 3			
TST	0.618	0.370–1.032	0.0658
SBP_NIght	1.028	0.987–1.070	0.1852
Model 4			
TST	0.614	0.368–1.025	0.0619
SBP_Sleep	1.026	0.986–1.068	0.2090
Model 5			
PLMSI	1.040	1.003–1.079	0.0322
SBP_NIght	1.028	0.987–1.070	0.1864
Model 6			
PLMSI	1.041	1.004–1.0979	0.0298
SBP_Sleep	1.027	0.986–1.069	0.2006

Logistic regression models were built to clarify the relation between sleep parameters and systolic BP during night and sleep. The only significant factor influencing the values of systolic blood pressure in night and in sleep was PLMSI (respectively: OR 1.040; 95 % CI 1.003–1.079; *p* = 0.0322 and OR 1.041; 95 % CI 1.004–1.0979; *p* = 0.0298).

## Discussion

In this study, we compared patients with RLS to subjects without a sleep disorder. We found that patients with RLS had higher nocturnal systolic blood pressures and that there was no significant difference (no dip) between values of daytime (evening) and nighttime values of systolic blood pressure.

We have compared very similar groups of subjects, differing clinically in terms of the presence of RLS. Prevalence of symptoms of metabolic syndrome and cardiovascular morbidity was not significantly higher among patients with RLS, which remains in concordance with results of epidemiologic studies [4, 19, 20].

Our findings are in concordance with the results of studies showing a higher prevalence of cardiovascular risk factors, like hypertension or hypercholesterolemia in patients with RLS. Such a conclusion was drawn from both large population-based studies [4, 19–24] and small observational studies [9].

The patients with RLS in our study had significantly higher values of nocturnal systolic blood pressure compared to the controls. A number of published articles have described the connection between RLS and/or PLMS, and increases in both heart rate and blood pressure. For example, Manconi et al. found a significant increase in heart rate following PLMS [25]. Pennestri et al. found that in patients with RLS, PLMS are followed by a significant increase in blood pressure and that the increase is even greater after PLMS associated with a microarousal [13]. A similar observation was made by Siddiqui et al., who found a significantly higher increase in BP following PLMS, even when compared to the BP increase following a voluntary leg movement [14]. Recently, those observations were confirmed in healthy subjects [15]. These findings suggest that patients with PLMS experience frequent transient elevations of blood pressure through the night, leading to a persistently increased nocturnal blood pressure. This is consistent with our results, in which the subjects with RLS had significantly more PLMS and PLMS with arousals.

The higher values of nocturnal systolic BP found in patients with RLS may be related to their disturbed sleep architecture. Indeed, patients with RLS had longer sleep latency and longer time of wake after sleep onset (WASO) what may result in higher values of BP in night. It must be noted then that systolic BP measured separately for the period of sleep was also higher in the group of patients with RLS.

Surprisingly, significant differences between the groups were found only for values of systolic blood pressure. Values of diastolic blood pressure were higher in the RLS group but that difference did not reach statistical significance. Data published so far showed that RLS and/or PLMS influence both systolic and diastolic BP, nevertheless some exceptions. Siddiqui et al. found that PLMS without arousal did not lead to a significant increase of diastolic BP [14]. Oh et al. compared patients with Parkinson’s disease and with/without RLS and described significantly higher values only of systolic BP in subjects with Parkinson’s disease and RLS [26]. Those data suggest that RLS and PLMS may be related to a sympathetic arousal system, but this relation may be too weak to influence diastolic blood pressure.

We also noted that unlike the control group, in which systolic blood pressure dipped at night, there was no significant change from diurnal to nocturnal systolic blood pressure in the group of patients with RLS. Notably, recently published data showed that RLS is an independent determinant for a non-dipping blood pressure pattern [9]. Furthermore, non-dipping blood pressure patterns were found to be related to higher RLS severity scores [27]. However, we had very limited data on the daytime BP values—it was measured from the beginning of the recording until the “lights-off” moment, so it reflects only evening BP values. Thus, this observation does not imply that patients with RLS have non-dipping BP patterns, though it does suggest that further study of this phenomenon is warranted. It is of note that in terms of non-dipping, the situation in RLS could be similar to that in obstructive sleep apnea syndrome (OSAS). There is a marked vasoconstriction and increase in BP following each episode of airway obstruction in OSAS [28], and those numerous surges of BP appearing throughout the night inhibit the nocturnal dip of BP. A role analogic to airway obstruction in OSAS could be played by PLMS in RLS. The final result is the same—repetitive increases in BP leading to a non-dipping pattern of BP. Non-dipping is considered an important risk factor of cardiovascular morbidity and organ damage due to hypertension [29] and therefore requires normalization. Normalization of BP is achieved with continuous positive airway pressure (CPAP) therapy in case of OSAS [30]. It can be speculated that in case of RLS, a similar effect could be achieved with suppressing PLMS, e.g., with dopaminergic treatment.

Another issue requiring further studies is the time course of changes of BP during the nights in patients with RLS. In our study, we have focused on overall nocturnal blood pressure while it would be of interest to observe whether values of BP differ between parts of nights dominated by slow-wave sleep and REM sleep. Such an analysis should become a part of upcoming projects.

The main limitation of our study, besides the relatively small group size, was that the patients were not drug-naïve in terms of antihypertensive drugs. Nevertheless, it was determined that the drug regimen was stable in the patients. The relatively low BP in both groups suggests that hypertension was well controlled in the patients, and the lack of difference in the daytime BP between the groups (despite the higher prevalence of hypertension in the RLS group) allows us to draw conclusions from the comparison of nocturnal blood pressure.

Participants of our study underwent only a single night in a sleep laboratory, and some changes in their sleep architecture could result from the first night effect. We wanted our results to be comparable with results from other groups, and published papers focusing on relation between RLS, PLMS, and BP were based upon single night observations [13–15, 31].

Another factor that could have influenced our results was presence of respiratory effort related arousals (RERAs). Patients with sleep-related breathing disorders were excluded from the study, and therefore, RERAs were not included in the analysis. It was believed that in groups selected in this way, respiratory events would not have much impact on values of blood pressure. Nevertheless, in must be remembered that RERAs, when present, have a significant impact on functions of cardiovascular system [32, 33]. The same must be noted on values of oxygen saturation: as patients with sleep-disordered breathing were not participating in this study, values of oxygen saturation were not included into analysis. The future projects aiming at disentangling the role of elements of sleep architecture in changes of BP should take RERA and values of oxygen saturation into account as relation between the latter factor and values of BP is well proven [34].

Our study showed that patients with RLS are at a higher risk of developing increased blood pressure during the night. This result does not establish a causal relation between RLS and hypertension, but some practical conclusions may be drawn from this study. First, blood pressure in patients with RLS should be monitored with 24-h ambulatory blood pressure monitoring (ABPM), as they may have normal values of blood pressure during the day but increased blood pressure at night. Second, a higher evening dose of the patient’s preferred antihypertensive drug should be considered in patients with RLS and hypertension, in an effort to prevent the increased nocturnal blood pressure. Future studies should concentrate on (1) clarifying the direction of the relationship between RLS and hypertension, (2) clarifying the relationship between RLS and metabolic disorders such as hypercholesterolemia, and (3) assessing the influence of RLS therapy on the dysregulated blood pressure.
